# Study protocol for The Emory 3q29 Project: evaluation of neurodevelopmental, psychiatric, and medical symptoms in 3q29 deletion syndrome

**DOI:** 10.1186/s12888-018-1760-5

**Published:** 2018-06-08

**Authors:** Melissa M. Murphy, T. Lindsey Burrell, Joseph F. Cubells, Roberto Antonio España, Michael J. Gambello, Katrina C. B. Goines, Cheryl Klaiman, Longchuan Li, Derek M. Novacek, Ava Papetti, Rossana Lucia Sanchez Russo, Celine A. Saulnier, Sarah Shultz, Elaine Walker, Jennifer Gladys Mulle

**Affiliations:** 1 Department of Human Genetics, Emory University School of Medicine, Whitehead Research Building, 615 Michael Street, Suite 300, Atlanta, GA 30322 USA; 2 Department of Pediatrics, Emory University School of Medicine, 1920 Briarcliff Road NE, Atlanta, GA 30322 USA; 3 Marcus Autism Center, 1920 Briarcliff Road NE, Atlanta, GA 30322 USA; 4 Emory Autism Center, 1551 Shoup Court, Atlanta, GA 30322 USA; 5 Department of Psychiatry and Behavioral Sciences, Emory University School of Medicine, 12 Executive Park Drive, 2nd floor, Atlanta, GA 30329 USA; 6 Department of Psychology, Emory University, 36 Eagle Row, Atlanta, GA 30322 USA; 7 Department of Epidemiology, Rollins School of Public Health, Emory University, Atlanta, GA 30322 USA

**Keywords:** 3q29 deletion, Protocol, Deep phenotyping, Rare variant

## Abstract

**Background:**

3q29 deletion syndrome is caused by a recurrent hemizygous 1.6 Mb deletion on the long arm of chromosome 3. The syndrome is rare (1 in 30,000 individuals) and is associated with mild to moderate intellectual disability, increased risk for autism and anxiety, and a 40-fold increased risk for schizophrenia, along with a host of physical manifestations. However, the disorder is poorly characterized, the range of manifestations is not well described, and the underlying molecular mechanism is not understood. We designed the Emory 3q29 Project to document the range of neurodevelopmental and psychiatric manifestations associated with 3q29 deletion syndrome. We will also create a biobank of samples from our 3q29 deletion carriers for mechanistic studies, which will be a publicly-available resource for qualified investigators. The ultimate goals of our study are three-fold: first, to improve management and treatment of 3q29 deletion syndrome. Second, to uncover the molecular mechanism of the disorder. Third, to enable cross-disorder comparison with other rare genetic syndromes associated with neuropsychiatric phenotypes.

**Methods:**

We will ascertain study subjects, age 6 and older, from our existing registry (3q29deletion.org). Participants and their families will travel to Atlanta, GA for phenotypic assessments, with particular emphasis on evaluation of anxiety, cognitive ability, autism symptomatology, and risk for psychosis via prodromal symptoms and syndromes. Evaluations will be performed using standardized instruments. Structural, diffusion, and resting-state functional MRI data will be collected from eligible study participants. We will also collect blood from the 3q29 deletion carrier and participating family members, to be banked at the NIMH Repository and Genomics Resource (NRGR).

**Discussion:**

The study of 3q29 deletion has the potential to transform our understanding of complex disease. Study of individuals with the deletion may provide insights into long term care and management of the disorder. Our project describes the protocol for a prospective study of the behavioral and clinical phenotype associated with 3q29 deletion syndrome. The paradigm described here could easily be adapted to study additional CNV or single gene disorders with high risk for neuropsychiatric phenotypes, and/or transferred to other study sites, providing a means for data harmonization and cross-disorder analysis.

## Background

As new genomic technologies are increasingly deployed in clinical settings, novel syndromes are being discovered at an astonishing pace [1]. However, articulating the range of clinical phenotypes associated with these syndromes lags behind the rate of discovery, leaving patients and clinical caretakers frustrated. This frustration is especially manifest when syndromes are associated with later-onset neuropsychiatric phenotypes such as schizophrenia, anxiety disorder, or bipolar disorder, as many are [2].

One barrier to phenotypic description is the low frequency of these syndromes, which may limit any one clinic to observing a single patient with a given syndrome. Clinical research collaboratives, such as ClinVar [3] https://www.ncbi.nlm.nih.gov/clinvar/) and the Undiagnosed Diseases Network [1] (https://undiagnosed.hms.harvard.edu/), parent-led initiatives aided by social media (e.g., Cornelia de Lange Syndrome Foundation, http://www.cdlsusa.org/) and internet-based registries [4] (https://simonsvipconnect.org/) have all allowed patients with similar genetic mutations to come together, even when the mutations are rare in the population. Once a critical mass of patients is assembled, a standardized phenotyping protocol can be applied in a research setting [5, 6]. The resulting data are of critical importance in a clinical context to inform standards of care, shape expectations for patients and their families, and strengthen the relationship between families affected by these disorders and the clinicians treating them. These data are also useful for research, to inform mechanistic studies and cross-disorder comparison. Here we describe a comprehensive and systematic phenotyping protocol we have developed for use in our recently launched study to describe the phenotypes spectrum associated with 3q29 deletion syndrome.

First described in 2005, 3q29 deletion syndrome is caused by a recurrent, typically de novo, 1.6 Mb heterozygous deletion on chromosome 3, with an estimated incidence of approximately one in 30–40,000 births [7]. The 3q29 deletion is associated with a range of neuropsychiatric phenotypes, including elevated risk of autism spectrum disorders (ASD) [8], intellectual disability [9–11], and anxiety disorder [12]. It is also associated with a 40-fold increased risk for schizophrenia [13, 14], which may make it the single-largest molecular risk factor for schizophrenia [7].

In addition, the neuropsychiatric phenotypes associated with the 3q29 deletion appear to emerge well below the average age of risk [12]. For example, in the largest published systematic study of 3q29 deletion carriers to date, Glassford and colleagues found that 28% of 44 respondents to an online registry report at least one psychiatric diagnosis. This sample was largely pediatric (the average age of deletion carriers in this study was 11.5 years old), suggesting that more neuropsychiatric illness may manifest as this cohort moves through the age at risk [12]. Thus, the true prevalence of neuropsychiatric manifestations in 3q29 deletion syndrome may be underreported. Consistent with this notion, anecdotal reports from parents suggest that some conditions (such as ASD and anxiety disorder) are not routinely assessed in individuals with 3q29 deletion syndrome. Furthermore, additional neurodevelopmental consequences associated with schizophrenia and other neuropsychiatric disorders are not well described, the degree of heterogeneity is undocumented, and the scope of physical and medical manifestations is unclear. Direct clinical evaluation can help to address these knowledge gaps.

The present project aims to determine and quantify the behavioral and clinical phenotype of 3q29 deletion syndrome in children and adults along four dimensions, selected because of their prior association with the syndrome: anxiety, cognitive ability, autism symptomatology, and elevated prodromal features and/or psychosis. Further, we seek to document physical attributes and comorbid medical symptoms and evaluate brain structure and function. In doing so, we document a systematic approach to deep phenotyping work in 3q29 deletion syndrome and lay a foundation for subsequent studies examining the relationship between behavioral and molecular phenotypes aimed at understanding the underlying mechanisms of schizophrenia and other associated neuropsychiatric disorders.

## Method

### Design

The present study is an ongoing prospective investigation into the behavioral and clinical phenotype associated with 3q29 deletion syndrome. A biospecimen (blood) is also collected from the 3q29 deletion carrier and any participating family members and banked for use in subsequent studies investigating the molecular mechanism of the disorder. Additionally, each deletion carrier completes a medical exam to evaluate medical history and current physical attributes, and an MRI scan to examine brain structure and function.

### Participants & setting

Study participants are individuals with 3q29 deletion and their family members. Participants are eligible if the carrier has a clinically confirmed diagnosis of the 3q29 deletion, is age 6 or older, is fluent in English, and the carrier and family are willing to travel to Atlanta, GA for assessment.

All evaluations take place at the Marcus Autism Center in Atlanta, GA, an affiliate of Children’s Hospital of Atlanta (CHOA), which has facilities designed for working with young people with a range of abilities and needs. The MRI scan takes place at the Center for Systems Imaging Core (CSIC) at Wesley Woods, which is part of Emory Healthcare.

Select self-report questionnaires are administered online prior to the visit using the REDCap web application. REDCap is approved for use in research environments to build and maintain online surveys and databases (https://www.project-redcap.org).

### Recruitment

Participants are recruited from the 3q29 registry (3q29deletion.org), which has been previously described [12], and from the 3q29 private Facebook community page, initiated and maintained by the families. The study is also described on the project website (http://genome.emory.edu/3q29/). We anticipate a final sample size of 30 individuals will be characterized. This sample size was based on feasibility of recruitment as well as statistical power: as our primary analysis, we will describe the presence and severity of phenotypes, and report whether these phenotypes are elevated in 3q29 deletion carriers compared to expected age-specific population estimates. Our sample size of 30 is well powered for this analysis as it has 80% power to detect an effect size half the magnitude of the phenotype’s standard deviation in the sample after applying a (conservative) Bonferroni correction for multiple testing.

When a family expresses interest in the study, they are sent copies of the study consent via email. A study team member contacts the family by phone to review the consent, answer questions, and confirm eligibility. During this initial phone screening, the team member verifies the accuracy of information from the registry (e.g., child diagnosis, age) and completes a brief eligibility screening for MRI. A hard copy of the consent and a medical record release form is mailed to the family with a postage paid return envelope. A signed consent and confirmation of diagnosis are required prior to the visit.

Within 4 weeks of the visit date, eligible participating families are contacted by a member of the MRI team to conduct a detailed eligibility screening for MRI. Participants with a contraindication for MRI are excluded from the MRI portion of the study.

### Visit protocol

#### Measures

Table 1 summarizes key clinical and behavioral measures.

**Table 1:** Study Measures by Schedule and Domain Assessed

Domain	Age	Measure
Anxiety	≤17 Years	*Anxiety Disorders Interview Schedule for DSM –IV (ADIS-IV) * Child Interview Schedule^a^ Parent Interview Schedule
	≥18 Years	*Kiddie Schedule for Affective Disorders and Schizophrenia (K-SADS) or Structured Clinical Interview for DSM-V* --*Research Version* (SCID-5-RV) - Module F^f^
Autism	All	*Autism Diagnostic Interview - Revised* * (ADI-R)*
	All	*Autism Diagnostic Observation Schedule, 2nd ed. * *(ADOS-2)* Modules 1, 2, 3, or 4
Cognitive Ability & Adaptive Function	≤17 Years	*Differential Ability Scales, 2nd ed. (DAS-II)*
	≥18 Years	*Wechsler Abbreviated Scale of Intelligence, 2nd ed. (WASI-II)*
	All	*Vineland Adaptive Behavior Scales, 3rd ed., Parent/Caregiver Form* ^b^
	≤18 Years > 18 Years	*Behavioral Rating Inventory of Executive Function*,* 2nd ed. (BRIEF-2)* ^b^ *Behavioral Rating Inventory of Executive Function*-*Adult Version (BRIEF-A)* ^b^
	All	*Beery-Buktenica Test of Visual Motor Integration, 6th Edition (VMI-6)*
Prodromal Symptoms & Psychosis	≥8 Years All	*Structured Interview for Psychosis-Risk Syndromes (SIPS)* Pre-interview^b, c, d^
	≥18 Years	*Structured Clinical Interview for DSM-V* --*Research Version* (SCID-5-RV) - Module B/C
General Psychopathology		*Kiddie Schedule for Affective Disorders and Schizophrenia* (K-SADS) ^e^ Child Interview^a^ Parent Interview Cross Cutting Survey^b, c, d^ Pre-interview^b, d^
	≤21 Years^f^ ≤21 Years^f^ All All	
	≥ 22 Years	*Structured Clinical Interview for DSM-V* --*Research Version* (SCID-5-RV) - Modules A, D, G, H, I, K
Anthropomorphic Measures/Medical Exam	All	Study generated Medical History Form and Vitals & Review of Systems-based Intake Form
Magnetic Resonance Imaging (MRI)	All	See MRI description in Methods section
Family Demographics^b^	All	Study generated Demographics Form

^a^The child interview portion is administered based upon the child’s capacity to engage in the interview. ^b^Administered prior to visit online via REDCap or publisher web application (BRIEF-2, BRIEF-A, Vineland-3). ^c^Completed by 3q29 deletion carriers when appropriate. ^d^Administered to all participants prior to visit to guide clinicians in identifying areas of focus during interview. ^e^To avoid overlap with the ADIS and ADI-R/ADOS, the anxiety and autism sections of the KSADS are omitted. ^f^Participants 18–21 years are administered the KSADS (including anxiety section) in lieu of the SCID-V-R, if level of communication abilities and emotional status suggest the need for parental perspective to ensure a thorough evaluation

##### Anxiety

Participants 18 years and younger are administered the *Anxiety Disorders Interview Schedule for DSM-IV (ADIS-IV) Child and Parent versions* [15]*,* which consist of clinician-administered, semi-structured interviews that assess the child and parent’s perception of the presence, duration, and degree of daily interference of specific anxiety symptoms. Anxiety symptoms are evaluated based on the Diagnostic and Statistical Manual of Mental Disorders – IV (DSM-IV). These comprehensive interviews assess all anxiety disorder diagnoses observed in young children. Moreover, interviewers are trained to reliability and are knowledgeable about the distinction between autism symptomatology and DSM-IV anxiety disorders. The child interview portion is administered based upon the child’s capacity to engage in the interview. If the child’s communication abilities and emotional status are not adequate to participate in the interview, it is omitted. For participants over 18 years of age, anxiety is assessed using the *Kiddie Schedule for Affective Disorders and Schizophrenia (K-SADS) or Structured Clinical Interview for DSM-5 Disorders (SCID-5)-Module F* (described subsequently in General Psychopathy section) based upon participant communication abilities.

##### Autism Spectrum disorder

The *Autism Diagnostic Interview, Revised (ADI-R)* [16] and *Autism Diagnostic Observation Schedule, Second Edition (ADOS-2)* [17] are used to help evaluate for Autism Spectrum Disorder (ASD). The ADI-R is a semi-structured comprehensive parent/caregiver interview designed to evaluate early developmental history and current and lifetime presentation of autism symptomatology.

The *ADOS-2* is a diagnostic, semi-structured clinical assessment that directly observes for behaviors associated with ASD in the areas of social communication, play and interaction, and restrictive and repetitive behaviors. It consists of a clinical procedure that places a child in unstructured, social and playful situations. During this assessment, the individual receives no instructions or guidelines indicating how to respond. In this way, a sample of naturalistic, social and communicative behaviors is obtained. The ADOS-2 consists of modules based upon age and language level: nonverbal or minimally verbal (Module 1), uses words and phrases (Module 2), speaks in complex sentences (e.g., connect ideas using “and” or “but”; Module 3), and verbally fluent older adolescents or adults (Module 4).

In anticipation of ADOS-2 and ADI-R administration, each participant’s 3q29 Registry data from four parent-report screening measures are downloaded, scored, and shared with clinicians: *Achenbach Behavior Checklist (CBCL/ABCL)* [18–20], *Social Responsiveness Scale-2* (SRS-2) [21], *Social Communication Questionnaire* (SCQ) [22], and *Autism Spectrum Screening Questionnaire* (ASSQ) [23]. The Achenbach checklists are commonly used measures to assess a range of social, emotional, and behavior problems. The SRS-2, SCQ, and ASSQ are widely used screening tools to assess aspects of the social and communication impairments typically associated with ASD.

##### IQ and adaptive behavior

Overall cognitive ability is assessed using the *Differential Ability Scales* – 2nd edition (DAS-II; 6–17 years) [24] or *Wechsler Abbreviated Scale of Intelligence, Second Edition* (WASI-II; 18 years and older) [25]. Along with cognitive ability, deficits in adaptive behavior are required for diagnosis of intellectual disability. Adaptive behavior, defined as the performance of day-to-day activities that are necessary for self-care and to get along with others, is assessed using *The Vineland Adaptive Behavior Scales, Third Edition, Parent Form* (Vineland-3) [26].

The DAS-II assesses cognitive skills in three domains: Verbal Reasoning, Nonverbal Reasoning, and Spatial Reasoning. In addition, a General Conceptual Ability Score (GCA) is reported. The WASI-II provides a brief but reliable measure of cognitive ability in the areas of Verbal Comprehension and Perceptual Reasoning [25], with an overall Full-Scale IQ, and verbal and performance IQs obtained.

The *Vineland-3* is a standardized parent interview that assesses skills an individual does independently on a daily basis in the areas of Communication, Daily Living Skills, Socialization, and Motor Skills. For the present study, the Vineland-3 is administered and scored using the Pearson q-Global online web application available through the publisher.

##### Visual-motor ability

Visual and motor abilities are evaluated using the three developmental tests of the *Beery-Buktenica Developmental Test of Visual-Motor Integration* – 6th edition (VMI-6) [27]. Together these three tests require participants to copy (Visual Motor Integration), identify (Visual Perception), and trace (Motor Coordination) each of 28 abstract designs of increasing complexity.

##### Executive function

Executive functions are assessed using the parent/informant forms of the *Behavioral Rating Inventory of Executive Function, 2nd edition* (BRIEF-2) [28] for participants up to 18 years old or the *Behavioral Rating Inventory of Executive Function- Adult Version (BRIEF-A)* [29] for participants over 18 years old. These inventories take 10 min (BRIEF-2) to 15 min (BRIEF-A) to complete and ask the informant to rate child behaviors associated with self-control and problem-solving skills along nine dimensions of executive functioning: inhibiting distractions, self-monitoring, shifting, emotional control, initiation, working memory, planning, organization, task monitoring. Scores within and over a specific threshold are considered “At Risk” or “Clinically Significant,” respectively. For the present study, the BRIEF-2 and BRIEF-A are administered and scored using the PARiConnect online web application available through the publisher.

##### Prodromal symptoms and psychosis

The *Structured Interview for Psychosis-Risk Syndromes (SIPS)* [30] assesses the presence, duration, and severity of subthreshold symptoms of psychosis. The SIPS is a reliable and valid semi-structured interview used to assess prodromal symptoms of psychosis and to determine if individuals meet criteria for an Attenuated Psychotic Syndrome, which is assessed across five different positive symptom domains: unusual thought content/delusional ideas, suspiciousness/persecutory ideas, grandiose ideas, perceptual abnormalities/hallucinations, and disorganized communication. This measure also yields ratings for six negative, four disorganized, and four general psychiatric symptoms. The SIPS is one of the primary instruments used in the North American Prodromal Longitudinal Study (NAPLS), enabling cross-comparison between these two studies [31].

Prior to the visit, the pre-interview questions for the SIPS are sent via REDCap to the parents and 3q29 deletion carrier (if over 18 years, and able to complete questionnaires). The SIPS-pre-interview questions take about 15–20 min to complete and ask about general medical and psychiatric history, and current thoughts, feelings, and perceptual experiences.

##### General psychopathology

The *Kiddie-Schedule for Affective Disorders and Schizophrenia (K-SADS)* [32] assesses general psychopathology among participants younger than 18 years. This semi-structured interview assesses both the child and parent’s perception of the presence, duration, and degree of daily interference of symptoms associated with affective and psychotic disorders. Symptoms are evaluated based on the *Diagnostic and Statistical Manual of Mental Disorders – V* (DSM-V) used by psychologists and other mental health providers to differentially diagnose affective or psychotic symptoms that interfere with daily functioning. Just as with the ADIS, the child interview portion is administered based upon the child’s capacity to engage in the interview.

The KSADS cross-cutting survey and pre-interview questions are sent to the parents and 3q29 deletion carriers (if applicable) before the visit via REDCap. Together the questionnaires take about 25–35 min and ask about reactions to experiences (cross-cutting) and general behavior and development (pre-interview).

For participants 18 years and older, general psychopathology, including anxiety, is assessed using the KSADS (if level of communication abilities and emotional status suggest the need for parental perspective to ensure a thorough evaluation) or *Structured Clinical Interview for DSM-5 Disorders Research Version (SCID-5-RV)* [33]. The SCID-5-RV is a semi-structured interview that assesses an individual’s perception of the presence, duration, and degree of daily interference of symptoms associated with various psychological disorders. Symptoms are evaluated based on the *Diagnostic and Statistical Manual of Mental Disorders – Fifth Edition* (DSM-5) used by psychologists and other mental health providers to differentially diagnose disorders including affective, anxiety, and psychotic disorders that interfere with daily functioning. Modules administered include: A (mood episodes), B/C (psychiatric disorders), D (mood disorders), F (anxiety), G (obsessive-compulsive and related disorders), H (sleep disorders), I (feeding and eating disorders), and K (externalizing disorders, including ADHD).

##### Anthropomorphic measures/medical exam

The medical exam is conducted by trained clinical geneticists (RS and MG) to assess medical history (e.g., birth weight, feeding problems), evaluate physical stature (e.g., height, weight, BMI, head circumference, Tanner Stage), and take high resolution photographs for rating by a dysmorphologist to determine whether a characteristic facial phenotype exists. A detailed medical history is completed online by the parent prior to the visit using the REDCap Survey feature. The clinical geneticists review this with the families in person at the study visit. Parents are also asked to sign a medical records release, which is used to request participant medical records, including growth trajectories and detailed medical history.

##### MRI

MRI data are collected on a Siemens Magnetom Prisma 3 T scanner at CSIC using a 32-channel head coil. High-resolution structural MRI, diffusion weighted imaging, and resting-state functional MRI data are acquired.

##### Structural MRI

T1-weighted and T2-weighted high-resolution structural images are acquired. T1-weighted images are acquired using a T1-weighted 3D MPRAGE sequence with the following parameters: TE = 2.24 ms, TR = 2400 ms, flip angle = 8^o^, matrix = 320 × 320, FOV = 256x256mm, 208 sagittal slices, 0.8 mm isotropic resolution, bandwidth = 210 Hz/pixel. A GRAPPA factor of 2 is used with no phase oversampling and two repetitions. The total scan duration is 13 min, 16 s. A 3D T2-weighted Sampling Perfection with Application optimized Contrast using different angle Evolutions (SPACE) sequence is employed. Details of the protocol are: TE = 563 ms, TR = 3200 ms, bandwidth = 745 Hz/pixel, FOV = 256 × 240 × 256 mm^3^, sagittally acquired, 0.8 mm isotropic resolution. A GRAPPA factor of 2 is used with no phase oversampling and two repetitions. The total scan duration is 11 min.

##### Diffusion weighted imaging (DWI)

DWI data are acquired using a high-angular-resolution-diffusion imaging (HARDI) protocol^96^ and multiband technique to reduce scan time while still acquiring data with high spatial and angular resolution^97,98^. Imaging parameters are: TR = 3222 ms, TE = 89.2 ms, multiband factor = 4, FOV = 212×184mm^2^, matrix = 106×97, b = 0, 1000, 2000s/mm^2^, 128 diffusion directions evenly distributed on the two shells, spatial resolution = 2 mm isotropic, 66 slices covering the whole brain, for a total of 10 averages of b0s. B0s in two shells have opposite phase encoding directions for removing susceptibility-related distortion present in dMRI^68^. Total scan duration is 8 mins.

##### Resting-state functional MRI

Resting-state functional MRI data are acquired using a multiband T2*-weighted EPI imaging sequence with the following parameters: TE = 33 ms, TR = 720 ms, flip angle = 53^o^ to match Earnest angle, matrix = 84 × 84, FOV = 210 × 210 mm, spatial resolution = 2.5 mm isotropic, 54 slices covering the whole brain, bandwidth = 2290 Hz/pixel, echo spacing = 0.58 ms. Data are collected across two runs, for a total of 1140 volumes. Total scan duration is 14 min, 30 s*.* 20 volumes of fMRI data are collected in the opposite phase encoding direction at the end of each fMRI run to correct for susceptibility-related distortion^94^.

##### Family demographics

A study-designed demographics questionnaire is used to gather information about the participant and family, including family size, race/ethnicity, parent education and employment, and household income. The questionnaire takes about 5 min to complete and is sent to the parent prior to the visit using the REDCap survey feature.

### Procedures

#### Visit preparation

Visits are scheduled at least 2–3 months in advance to allow time to coordinate clinical team availability and make travel arrangements. To prepare, within a month of the visit date, an email link to the pre-visit self-report questionnaires (see Table 1) is sent from REDCap. When appropriate, participants over 18 years old are also asked to complete applicable questionnaires (see Table 1). The questionnaires include the family demographic form, pre-interview questions for the SIPS and KSADS, KSADS Cross Cutting survey, medical history form, and a brief survey of prodromal symptoms [34] (if not already completed in the registry). Once a family completes the questionnaires, they are sent links to complete the *Vineland-3* and *age-appropriate BRIEF* (if applicable).

Families are also invited to provide any current Individualized Education Plans (IEPs) and medical, psychological, or psycho-educational testing reports that could help the team in assessing the participant.

Approximately one week prior to the visit, clinical team members receive the participant *dossier* that includes questionnaire data and reports provided by the family to help prepare for the visit. Also included are select participant data from behavioral measures completed as part of the 3q29 Registry [5]: CBCL, SRS-2, SCQ, and ASSQ, survey of prodromal symptoms [34].

#### Visit execution

**Fig. 1:**
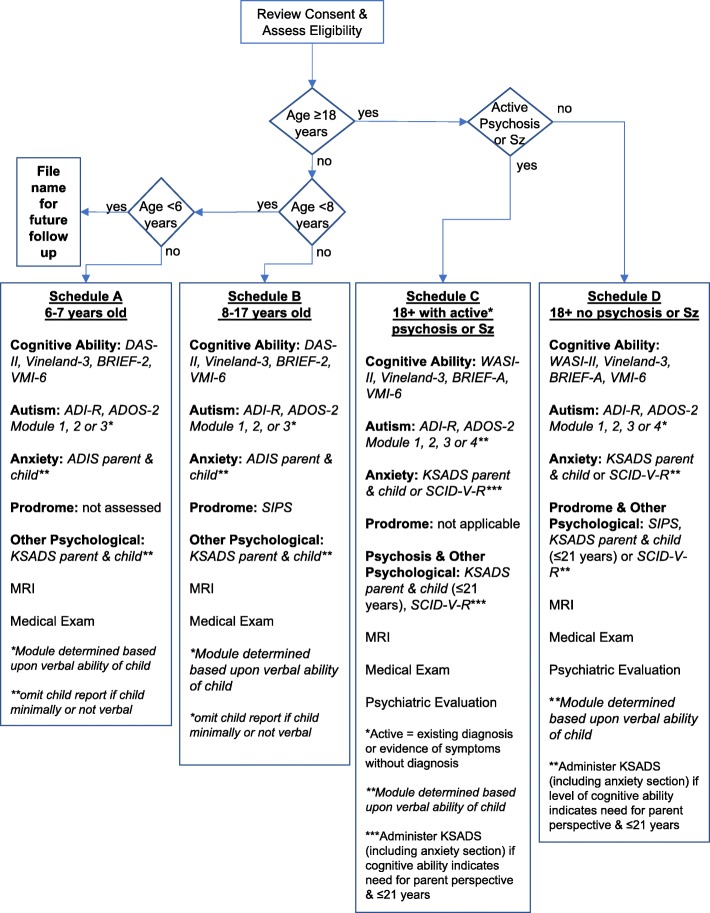
Process flow diagram of the study protocol for phenotyping of 3q29 deletion syndrome. Assessment administration is determined based upon participant age, verbal ability, and evidence of active psychosis (defined as existing diagnosis of schizophrenia or evidence of psychosis without diagnosis). For participants under 18 years, the anxiety and autism sections of the KSADS are omitted to avoid overlap with the ADIS and ADI-R/ADOS-2. In this age group, child versions of the KSADS and ADIS are administered when the child’s communication ability and emotional status allow engagement in the interview. Participants 18–21 years are administered the KSADS (including anxiety section) in lieu of the SCID-V-R, if their level of communication ability and emotional status suggest the need for parental perspective. Sz = Schizophrenia

Figure 1 summarizes the study protocol. Participant age, verbal ability, and presence of active schizophrenia or psychosis are used to determine testing schedule and measures administered. For example, a verbally fluent 8-year-old child would complete Visit Schedule C; a 21-year-old with no evidence of active psychosis would complete Visit Schedule F.

Responses in the registry and confirmed during the initial phone screening are used to determine verbal ability (based upon ADOS-2 requirements, described subsequently) and presence of schizophrenia or psychosis. Sometimes conversation with the family suggests the presence of unconfirmed or undiagnosed psychosis for participants under 18 years old. In these cases, the visit schedule is modified to include individual psychiatric evaluation by the team psychiatrist (JFC).

Testing takes place over two week days. To ensure informed consent prior to testing, a study team member reviews the consent form in detail with the family and answers questions upon their arrival at the testing center. When applicable, child assent is obtained. With parent or participant permission, behavioral assessments are video and audiotaped for future reference. The medical history is audiotaped.

##### Blood sample collection

All participants and family members are asked to provide 4–5 vials (about 3 tablespoons) of blood from a vein in the arm. Blood is drawn by a trained pediatric phlebotomist (J J-D). To avoid biasing the results of assessments due to a negative experience with a blood draw, the blood sample is scheduled towards the end of the first day’s assessments. Remote blood draws are arranged locally for family members who are unable to travel to Atlanta for the evaluation.

##### MRI

The MRI scan lasts 30–120 min with breaks as needed. During the scan, participants are asked to view video clips of people talking and interacting. An 8-min segment is also required where the participant simply lays still with no video input.

#### Visit wrap-up

At the end of the second visit day, the clinical team convenes privately to discuss results, impressions, and recommendations. Following this clinical case conference, the team meets with the family to share their impressions: preliminary results of evaluations, the relative strengths and challenges of the person with 3q29, and any recommendations for ongoing care. Within a month of the study visit, the family also receives a comprehensive written report summarizing the results from each evaluation, and study contact information is provided in case there are concerns or questions. Following the visit, families are sent a feedback questionnaire via REDCap to enable further improvement of our process.

Family lodging and travel expenses are arranged and paid for by the study, and each participating family member receives an honorarium for the time involved in participation.

### Data management and analysis

Family information, study questionnaires, medical exam data, photos, and recordings are stored in REDCap. The standardized study instruments are modeled in a local software system housed at the Marcus Autism Center, called DEX (Marcus Data Exchange). Modeling refers to the creation of the data structure for a given assessment/form and the accompanying platform to facilitate data entry (i.e., virtual form). The instruments are modeled following the National Database for Autism Research (NDAR)'s data dictionary guidelines, for future export from DEX into NDAR. All data entered into REDCap are double checked for accuracy. DEX follows a dual data entry system with automatic data checks, to ensure accurate data entry.

## Discussion

The present paper describes the study protocol for a prospective study of the behavioral and clinical phenotype associated with 3q29 deletion syndrome. Systematic assessment of neuropsychiatric phenotypes has several advantages. First, consistent evaluation of the neurodevelopmental effects of the deletion can be used to establish standards of clinical care, including recommendations for early screening and intervention services, which may affect both short and long-term outcomes and management of the disorder, and are of high importance to families. It also establishes a baseline against which to measure the effects of any future therapeutic interventions.

Second, standardized assessment of all participants along the same four dimensions permits evaluation of whether there is an association among behavioral phenotypes or whether the phenotype arises as the result of a “core lesion” that primes the individual to develop a specific outcome. For example, an associated phenotypes hypothesis predicts that the presence and severity of each dimension of the phenotype is associated with the others (e.g., lower IQ is associated with higher anxiety and more compromised social functioning, which in turn are associated with more prodromal symptoms). Alternatively, a core lesion hypothesis suggests that an individual is primed to develop schizophrenia, autism, or anxiety, but that variable external forces at the behavioral or molecular level contribute to the specific phenotype. Evaluating these competing hypotheses provides important insights into potential mechanisms affecting the emergence of behavioral phenotypes in 3q29 deletion and other syndromes.

A third advantage of deep phenotyping work is that it can be leveraged for future studies aimed at linking behavioral and molecular data (neuronal phenotypes). Indeed, cellular and molecular data gathered in this study are being used to examine hypotheses including the effect of diploid status on the 3q29 neuronal phenotype, correlations between behavioral severity and neuronal morphology or function, and the presence of molecular phenotypes in inhibitory neurons. We will also have the opportunity to assess polygenic influences [35] and genetically dissect the relative contributions of interval genes to the multiple phenotypes expressed by carriers [36].

Finally, standardized assessment and systematic data capture of manifestations in 3q29 deletion syndrome, operationalized according to the protocol described here, allows for cross-comparison with other rare genetic syndromes, such as 16p11.2 duplication [5] and 22q11.2 deletion [37], where nearly identical instruments are deployed. As new syndromes with high risk for neuropsychiatric phenotypes are identified, it will become even more important to have harmonized systems for phenotypic evaluation. The protocol described here is one way forward.

Beyond the contributions of this study to describing the behavioral phenotype of 3q29 deletion and understanding its molecular and genetic basis, this study serves as an opportunity for family engagement, support, and education. For example, families report appreciating the opportunity to connect with professionals who are familiar with 3q29 deletion, its causes, and manifestations. Moreover, evaluations of the scope used in this study can be cost prohibitive for families seeking to have such testing independently. Although study evaluations are for research purposes, the detailed clinical report of the evaluations and follow up recommendations provided by our team of clinicians can support the development of interventions, inform IEPs, or help individuals qualify for services.

Continued evaluation of the neurodevelopmental effects of the deletion require that future studies follow individuals over time to document aspects of the phenotype as they change (e.g., low BMI and failure-to-thrive) or emerge over time (e.g., schizophrenia). Such work is strengthened by our efforts to build partnerships between families and researchers, as well as establish an international network of research collaborators. With the paradigm presented here, we hope to advance our understanding of 3q29 deletion syndrome, which may in turn serve as an entry point into a general molecular mechanism of neuropsychiatric phenotypes such as schizophrenia and autism.

## Abbreviations

ADHD: Attention deficit hyperactivity disorders
ADI-R: *Autism diagnostic interview schedule, revised edition*
ADIS-IV: *Anxiety disorders interview schedule for DMS-IV*
ADOS-2: *Autism diagnostic observation schedule, 2nd edition*
ASD: Autism spectrum disorder
BRIEF-2 (BRIEF-A): *Behavioral rating inventory of executive functions, 2nd edition (adult version)*
CBCL/ABCL: *Child (Adult) behavior checklists*
CHOA: Children’s Hospital of Atlanta
CNV: Copy number variant
CSIC: Center for Systems Imaging Core
DAS-II: Differential Ability Scales, *2nd edition*
DSM-IV (DSM-V): *Diagnostic and statistical manual of mental disorders,* *4th edition (5th edition)*
K-SADS: *Kiddie-schedule for affective disorders and schizophrenia*
NAPLS: North American Prodrome Longitudinal Study
NRGR: NIMH Repository and genomics resource
SCID-5: Structured clinical interview for DSM-5 disorders
SCQ: *Social communication* *questionnaire*
SIPS: *Structured interview for psychosis-risk syndromes*
Sz: Schizophrenia
VMI: *Beery-Buktenica developmental test **of visual-motor integration*, 6th edition
WASI: *Wechsler abbreviated scale of intelligence, 2nd edition*
